# From antenna to reaction center: Pathways of ultrafast energy and charge transfer in photosystem II

**DOI:** 10.1073/pnas.2208033119

**Published:** 2022-10-10

**Authors:** Shiun-Jr Yang, Eric A. Arsenault, Kaydren Orcutt, Masakazu Iwai, Yusuke Yoneda, Graham R. Fleming

**Affiliations:** ^a^Department of Chemistry, University of California, Berkeley, CA 94720;; ^b^Molecular Biophysics and Integrated Bioimaging Division, Lawrence Berkeley National Laboratory, Berkeley, CA 94720;; ^c^Kavli Energy Nanoscience Institute at Berkeley, Berkeley, CA 94720;; ^d^Department of Plant and Microbial Biology, University of California, Berkeley, CA 94720

**Keywords:** photosystem II, ultrafast spectroscopy, energy transfer, charge separation

## Abstract

The photosystem II core complex (PSII-CC) is the smallest subunit of the oxygenic photosynthetic apparatus that contains core antennas and a reaction center, which together allow for rapid energy transfer and charge separation, ultimately leading to efficient solar energy conversion. However, there is a lack of consensus on the interplay between the energy transfer and charge separation dynamics of the core complex. Here, we report the application of two-dimensional electronic-vibrational (2DEV) spectroscopy to the spinach PSII-CC at 77 K. The simultaneous temporal and spectral resolution afforded by 2DEV spectroscopy facilitates the separation and direct assignment of coexisting dynamical processes. Our results show that the dominant dynamics of the PSII-CC are distinct in different excitation energy regions. By separating the excitation regions, we are able to distinguish the intraprotein dynamics and interprotein energy transfer. Additionally, with the improved resolution, we are able to identify the key pigments involved in the pathways, allowing for a direct connection between dynamical and structural information. Specifically, we show that C505 in CP43 and the peripheral chlorophyll Chlz_D1_ in the reaction center are most likely responsible for energy transfer from CP43 to the reaction center.

## Significance

The photosystem II core complex (PSII-CC) is a photosynthetic complex that contains antenna proteins, which collect energy from sunlight, and a reaction center, which converts the collected energy to redox potential. Understanding the interplay between the antenna proteins and the reaction center will facilitate the development of more efficient solar energy conversion technologies. Here, we study the sub-100-ps dynamics of PSII-CC with two-dimensional electronic-vibrational spectroscopy, which connects energy flows with physical space, allowing a direct mapping of energy transfer pathways. Our results reveal a complex dynamical scheme which includes a specific pathway that connects CP43 to the reaction center. Resolving this pathway experimentally provides insights into the energy conversion processes in natural photosynthesis.

**Fig. 1.**
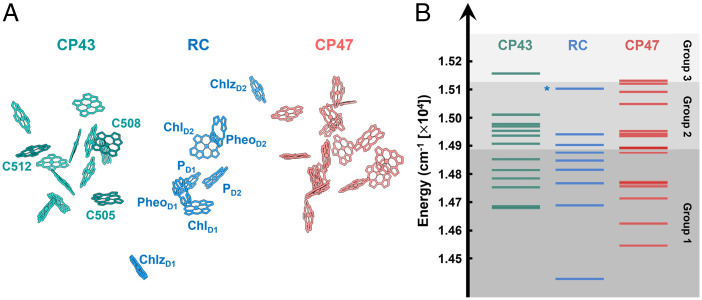
(*A*) Pigment arrangement of monomeric PSII-CC (whereas it is typically found as a dimer) depicted based on the cryoelectron microscopy structure (3JCU) reported by Wei et al. (10). The pigments of CP43, RC, and CP47 are shown in green, blue, and red, respectively. (*B*) Corresponding excitonic energy levels of monomeric PSII-CC color coded to match pigments in *A* (55–57). The gray shaded regions in the background represent the three groups based on similar characteristic dynamics. Note that the boundaries between the groups provide only a rough separation region as the dynamical behaviors change gradually along ω_exc_. The asterisk (for the RC state) indicates an optically dark state.

Photosynthesis is the process through which solar energy is converted into chemical energy (1–3). Photosystem II (PSII), a pigment–protein complex found in cyanobacteria, algae, and land plants, is the site of water splitting and is therefore crucial for photosynthetic function (4–6). It is connected with a large light-harvesting antenna system that collects solar energy and transfers the energy to the reaction center (RC), where charge separation (CS) occurs. Unlike the antenna system of purple bacteria that has a clear energy funnel, the PSII antenna system has a more complicated composition and a very complex energy landscape (4–7). These features allow for regulation that responds to rapid environmental fluctuations and protect the organisms in, for example, excess light, while maintaining highly efficient electronic energy transfer (EET) under optimal conditions (8). To understand the intricate interactions between the subunits that allow for the robustness of this photosynthetic system, the first step is to understand how the antenna system is connected to the RC. The PSII core complex (PSII-CC) is the smallest unit in which the RC is connected to the antenna proteins. It is a dimeric pigment–protein complex in which each monomer contains an RC and two core antenna proteins, namely, CP43 and CP47 (1, 7). These core antennas not only harvest solar energy but also act as the crucial bridge between the peripheral light-harvesting antenna system and the RC. Fig. 1*A* shows the pigment arrangement of the PSII-CC. The RC, consisting of the D1 and D2 branches, binds the following pigments: 1) two special pair chlorophyll *a* (P_D1_ and P_D2_), 2) two accessory chlorophyll *a* (Chl_D1_ and Chl_D2_), 3) two pheophytin *a* (Pheo_D1_ and Pheo_D2_), and 4) two peripheral chlorophyll *a* (Chlz_D1_ and Chlz_D2_) (9, 10). Despite the similarity between the D1 and D2 branches, CS occurs only along the D1 branch (11, 12). CP43, one of the two core antenna proteins, contains 13 chlorophyll *a* (Chls) and is located closer to the D1 active branch. CP47 contains 16 Chls and is located closer to the D2 branch (10). Together, these proteins provide highly effective EET and CS, which are key to the high quantum yield of CS in the RC.

Despite the importance of the PSII-CC, its early time dynamics is not fully understood—specifically the competition between EET and CS (5, 7). This is largely due to the highly congested excitonic manifold (Fig. 1*B*) and ultrafast EET timescales, which challenge ultrafast spectroscopic techniques. Two distinct models have been put forth to try to describe the function of the PSII-CC. These two models are the “exciton/radical pair equilibrium” (ERPE) model (13–17) and the “transfer-to-trap limited” (TTTL) model (18–22). An early fluorescence decay experiment (13, 14) suggested that rapid EET allows the excitonic states to reach an equilibrium between the core antennas and the RC before CS occurs (*k*_EET_ ≫ *k*_CS_), which is the basis for the ERPE model. This model was later supported by improved time-resolved fluorescence (15) and transient absorption experiments (16). However, a major discrepancy in this model arose with the measurement of the X-ray crystal structure of the PSII-CC (18). It was suggested that the large distances (center-to-center distance, >20 Å) between antenna and RC pigments resolved in the crystal structure would mean that ultrafast EET between the antenna proteins and the RC is unlikely. A model was then put forth that instead suggested that the EET from the core antenna to the RC is slow compared to CS (*k*_EET_ ≪ *k*_CS_), and therefore, the EET to the trap becomes a kinetic bottleneck (18). This TTTL model was later supported by transient infrared (IR) (19) and time-resolved fluorescence experiments (20, 21) as well as structure-based simulations (22). Additionally, Kaucikas et al. (23) performed a polarized transient IR experiment on an oriented single PSII-CC crystal. The decay of the polarization-dependent signature (50–100 ps) observed in their experiment suggests that equilibration between different subunits is slow, consistent with the TTTL model. However, it has been pointed out that satisfactory fitting of the spectral evolution to this model does not necessarily imply that it is correct (24, 25), especially as others have shown that the EET dynamics cannot be adequately described by a single hopping scheme (26, 27). A recent two-dimensional electronic spectroscopy (2DES) experiment (28) with improved time resolution has also revealed the existence of ultrafast EET (<100 fs) that was not predicted by theoretical calculations. In their work, Pan et al. (28) attributed the origin of this unexpectedly fast EET pathway to polaron formation. Vibronic effects on the ultrafast EET and CS dynamics of other photosynthetic proteins have also been discussed (29–38).

The lack of detailed understanding of the PSII-CC early time dynamics, in particular the EET between the core antennas and the RC, highlights the need for further experimental input with the ability to make specific assignments of the dynamical pathways. This, however, requires simultaneous high temporal and spectral resolution, which remains a challenge for ultrafast spectroscopic techniques. Here, we describe the application of two-dimensional electronic-vibrational (2DEV) spectroscopy (39–41) to the PSII-CC. The combination of both spectral dimensions provides an improved resolution that allows us to obtain much more detailed dynamical information in complex systems. The excitonic energy landscapes generated by electronic coupling in photosynthetic complexes, combined with site-dependent and charge state–dependent vibrational spectra, allow the resolution along both axes of 2DEV spectra to provide a direct connection between energetic space (via visible excitation) and physical space (via IR detection). This advantage has proven to be useful for the studies of dynamics in photosynthetic pigment–protein complexes (33, 40–45). Specifically, the resolution along the electronic excitation axis allows for the separation of the contributions from different pathways, while the resolution along the vibrational detection axis provides a way to identify the protein subunits or even specific states involved in the dynamics. As we will show, this unique feature of 2DEV spectroscopy provides insight into the complex dynamics of the PSII-CC.

In the following text, we will show that the sub-100-ps dynamics of the PSII-CC extracted from spinach are highly dependent on the excitation frequency range. The resolution along the detection axis allows different dominant dynamics to be identified. In addition, we will demonstrate how 2DEV spectroscopy allows us to connect the observed dynamics to specific excitonic states. This connection allows us to obtain a more specific pigment assignment for the EET pathways and therefore provides a more detailed understanding of the finely tuned interactions between the RC and the core antennas (specifically CP43, which is closer to the active D1 branch). We will conclude with a comparison between our results and the existing models in order to provide a path forward in the understanding of this critical photosynthetic component.

## Results

### 2DEV Spectra and IR Band Assignments.

**Fig. 2.**
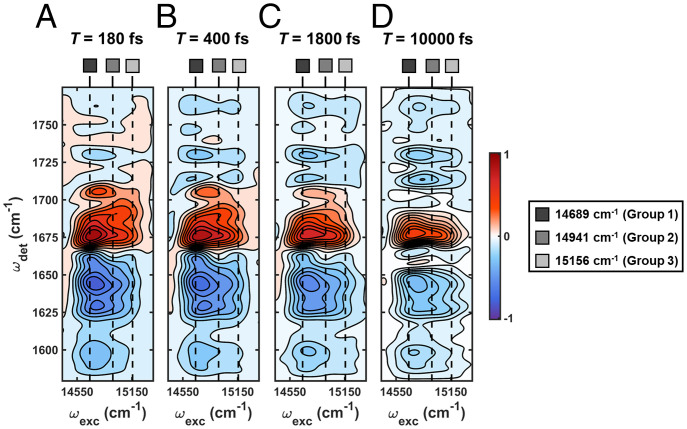
2DEV spectra at (*A*) *T* = 180 fs, (*B*) *T* = 400 fs, (*C*) *T* = 1,800 fs, and (*D*) *T* = 10,000 fs. Positive contours (red) indicate GSB features, and negative contours (blue) indicate PIA features.

Representative 2DEV spectra of the PSII-CC at 77 K are shown in Fig. 2. The excitation energy range was selected to cover the Q_y_ bands of the Chl and pheophytin (Pheo) chromophores in the PSII-CC. At early waiting times (Fig. 2 *A*–*C*, *T* = 180, 400, and 1,800 fs), the spectrum shows a vibrational structure (i.e., detection frequency [ω_det_]) that is highly dependent on excitation frequency (ω_exc_), while at much later waiting times (Fig. 2*D*, *T* = 10,000 fs), this ω_exc_ dependence vanishes. The convergence of the vibrational structure along the ω_exc_ axis as the waiting time evolves is a clear signature of population transfer in 2DEV spectroscopy (33, 42). Furthermore, the distinct spectral evolution along ω_det_ at different ω_exc_s is an indication that the pathway leading to the formation of the final state varies significantly depending on the initially populated levels. Without resolution along the ω_exc_ axis, the contributions from all of these pathways would be convoluted together, resulting in significant spectral congestion despite the improved frequency resolution afforded by IR detection. The simultaneous frequency resolution along both the ω_det_ and ω_exc_ axes afforded by 2DEV spectroscopy is critical for untangling the contributions from different pathways because the dispersal of the corresponding vibrational signatures along the ω_exc_ axis significantly eases the interpretation of the dynamics and states involved.

For the vibrational assignments, we rely on previous steady-state, transient IR and 2DEV experiments on the PSII-CC and its constituent parts (19, 45–52). These works have shown that the localized stretching modes of the 13^1^-keto (previously 9-keto) and 13^3^-ester (previously 10a-ester) carbonyl groups in the Chl and Pheo chromophores can serve as spatial proxies due to the sensitivity of these vibrations to the local environment (46–49). Although some of the vibrational assignments were reported for room temperature, we do not expect the effect of temperature on these modes to be significant, as these modes are highly local and are not anharmonically coupled to lower frequency modes. Therefore, the ω_det_ range in our experiment was selected to cover the carbonyl modes (spanning 1,575 to 1,775 cm^−1^) in order to distinguish between specific Chl and Pheo molecules throughout the PSII-CC. Three major bands are present in the 2DEV spectra, as follows: 1) the photoinduced absorptions (PIAs) around 1,610 to 1,670 cm^−1^, corresponding to the 13^1^-keto carbonyl group in the excited state; 2) the ground state bleach (GSB) around 1,670 to 1,710 cm^−1^, corresponding to the 13^1^-keto carbonyl group in the ground state; and 3) the PIA around 1,710 to 1,760 cm^−1^, corresponding to the 13^3^-ester carbonyl group. As a result of the environmental sensitivity of the frequencies of these modes (46–49), an additional vibrational structure is observed in the abovementioned spectral regions. Throughout the waiting time, the vibrational structure reveals key GSB peaks at 1,657 cm^−1^, 1,691 cm^−1^, and 1,706 cm^−1^ and PIA peaks at 1,654 cm^−1^, 1,660 cm^−1^, 1,711 cm^−1^, and 1,715 cm^−1^. The GSB at 1,691 cm^−1^ is exclusive to the Chls in CP43 (52), whereas the GSB at 1,706 cm^−1^ is specific to P_D1_ in the RC (51). The PIA around 1,654 cm^−1^ was found in both CP43 and CP47 (50, 52), whereas for the RC, a PIA feature is found at 1,660 cm^−1^ (45, 51). Progressive CS (Chl_D1_^+^Pheo_D1_^−^ → (P_D1_P_D2_)^+^Pheo_D1_^−^) in the RC can be tracked via the features at 1,657 cm^−1^ (GSB) and 1,660 cm^−1^ (PIA) as well as the redshift in the PIA feature at 1,715 cm^−1^ to 1,711 cm^−1^ (which specifically follows hole transfer from Chl_D1_^+^ to (P_D1_P_D2_)^+^, respectively) (45). All of the above peaks provide critical information for understanding the dynamics of EET and CS in the PSII-CC. It is also worth noting that the GSB specific to the Chls in CP47, at 1,686 cm^−1^ (51, 53), does not appear as prominently as the other features listed above, as it is most likely obscured by the strong GSBs of the RC and CP43 (specifically, the broad band around 1,677 cm^−1^, which originates from both the RC and CP43 pigments, and the 1,691 cm^−1^ peak, which originates exclusively from CP43 pigments). This, unfortunately, limits our ability to extract information about CP47. Consequently, while the presence of this exclusive CP47 marker can be found in the spectra to a limited extent, we focus the following discussion mostly on the interplay between CP43 and RC states for which clearer spectral markers are present.

### Global Analyses of 2DEV Spectral Slices.

The significant ω_exc_ dependence of the 2DEV spectra suggests that global analyses (54) should be performed on individual 2DEV spectral slices taken at fixed ω_exc_. The values of ω_exc_ were selected according to the excitonic levels of the RC based on the empirical Hamiltonian by Novoderezhkin et al. (55), whereas those of CP43 and CP47 are based on the work of Müh et al. (56) and Hall et al. (57), respectively (Fig. 1*B*). We chose to use different empirical models for the RC and the core antennas, CP43 and CP47, because recent works have shown that exciton-charge transfer (CT) mixing is critical to efficient CS in the PSII-RC (45, 55); therefore, it is important to include a CT state in the Hamiltonian describing the RC. However, to our knowledge, there is currently no Hamiltonian for the full PSII-CC that explicitly includes a CT state. We base the energies for the excitonic states of the PSII-CC on Hamiltonians constructed to describe the isolated constituents of the PSII-CC, leading to a concern that interprotein pigment couplings could alter the energy levels and spectra. However, the interprotein pigment couplings have been shown to be small (27, 58), and therefore, we expect the calculated excitonic energy levels still provide reasonable insight even without the consideration of the interprotein pigment couplings.

For the global analyses, the data were fitted with a sequential model. It is important to note that the sequential model does not fully reflect the actual dynamics of the PSII-CC due to the expected existence of reverse steps and multiple parallel pathways. This is to say that the results of the global analyses do not necessarily represent the evolution of unbranched, unidirectional processes. Rather, the results reflect reversible steps and the convolution of multiple pathways occurring on similar timescales. To address the former, we perform the experiment at 77 K—reducing the amount of reverse transfer. Carrying out the experiment at 77 K influences the overall dynamics of CP43 and CP47 transfer to the RC. According to the model of Raszewski and Renger (22), the EET from CP43 to the RC speeds up by roughly a factor of two from 300 K to 77 K (absolute time constants, 41 ps at 300 K and 26 ps at 77 K), whereas the EET from CP47 to the RC slows down by an order of magnitude (absolute time constants, 50 ps at 300 K and 360 ps at 77 K) (22). Additionally, Shibata et al. (26) analyzed the red shift observed in PSII-CC fluorescence spectra between 5 K and 77 K and reached a similar conclusion, i.e., EET from CP47 to the RC is blocked at 77 K. This phenomenon clearly limits the short time information we can extract from our 2DEV spectra for the EET from CP47 to the RC, and we focus the majority of the analysis on relaxation within CP43 and the EET from CP43 to the RC. To ease the degree of convolution, we have specifically preformed the global analysis as a function of ω_exc_—greatly reducing the number of parallel pathways that contribute to each of the obtained timescales. Even by doing so, there may still be numerous parallel processes contributing to a given timescale due to the likely excitation of multiple excitonic states at a given ω_exc_ (as the absorption frequency range of the excitonic states has a finite width). In order to further deconvolute the results of the global analyses, we rely on the spectral structure of the evolution-associated difference spectra (EADS) (54). The vibrational structure of the EADS provides a means to identify specific excitonic states contributing to the obtained timescales (based on the distinct vibrational frequencies of key pigments in the PSII-CC). For example, we can use the presence of the key vibrational marker of CP43 in the EADS to understand the relative contribution of EET between the core antenna and the RC to a given timescale, or similarly, we can track the markers for the CS species in the RC to understand the relative contribution of these pathways. The global analyses based on a simplified sequential model, therefore, serve to provide an understanding of the dynamics of the PSII-CC afforded by the dual-frequency resolution particular to 2DEV spectroscopy.

Based on the number of components required for fitting, we roughly separate the ω_exc_ axis (i.e., the excitonic states) into three main groups, as represented by the different shaded regions in Fig. 1*B*. We find that each group has its own characteristic dynamics. A closer inspection of the dynamics within a given group, however, does reveal some interesting differences, which we will later interpret to gain further insight into the complex interplay between the energetic and spatial landscapes of the PSII-CC. In this section, we will discuss the characteristic dynamics for each group, starting from the first group (lowest ω_exc_ range) and ending by the last group (highest ω_exc_ range).

**Fig. 3.**
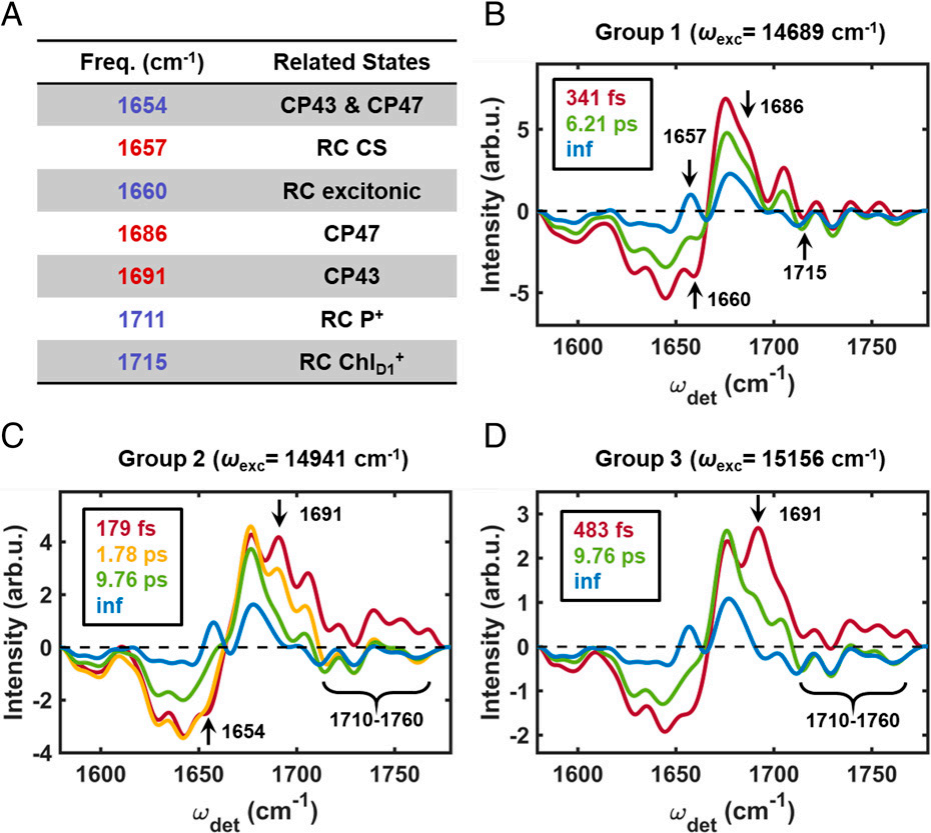
(*A*) Table of major IR peak assignments. The frequencies in red indicate GSBs and those in blue indicate PIAs. (*B*–*D*) EADS and corresponding timescales for a representative ω_exc_ from groups 1 to 3, respectively.

Fig. 3 *B*–*D* shows the results of the global analysis at representative ω_exc_s from each of the three groups. The evolution is interpreted such that the EADS evolves from one to the next with the associated time constant. The first group (Fig. 3*B*, representative ω_exc_ = 14,689 cm^−1^) requires three components to obtain a reasonable fit (with the last one being a nondecaying component, indicating the presence of a timescale beyond the duration of the experiment). Focusing on the key vibrational markers, the major spectral changes for both steps (evolution from red to green EADS and green to blue EADS) are found around 1,657 cm^−1^, 1,660 cm^−1^, and 1,715 cm^−1^. The gradual disappearance of the feature at 1,660 cm^−1^ and the concomitant growth of the peak at 1,657 cm^−1^ are very similar to the evolution observed in the 2DEV spectra of the isolated PSII-RC, as is the red shift of the feature observed at 1,715 cm^−1^ (45). Therefore, we assign the dominant dynamics for this group to rapid CS ((Chl_D1_^δ+^Pheo_D1_^δ−^)* → Chl_D1_^+^Pheo_D1_^−,^ ∼340 fs) followed by hole migration from Chl_D1_^+^ to (P_D1_P_D2_)^+^ (∼6.2 ps) (45). We also note that a shoulder around 1,686 cm^−1^ is found in both the first and the second EADS (Fig. 3*B*, red and green), which indicates the excitation of CP47 Chls at this ω_exc_. This feature undergoes a decay in both steps (Fig. 3*B*, evolution from red to green EADS and green to blue EADS), suggesting that EET occurs on similar timescales to the RC dynamics. The dynamics initiated in this frequency range are dominated by intraprotein dynamics.

For the second group (Fig. 3*C*, representative ω_exc_ = 14,941 cm^−1^), an additional component is needed to obtain reasonable fits. The structures of the EADS and the timescales are strikingly different from those of the first group—indicating distinct dynamics in this spectral region. For the first step, occurring on a ∼180-fs timescale, (Fig. 3*C*, evolution from red to yellow EADS), the peak at 1,691 cm^−1^ (specific to CP43) undergoes a significant decay but does not disappear completely. This decay is accompanied by a growth of the PIA region spanning 1,710 to 1,760 cm^−1^, indicating energy is transferred from a CP43 state to a new excited state(s). Without other clear spectral features, it is difficult to definitively assign the new state(s). In a previous 2DES experiment (28) and structure-based calculation (22), ultrafast EET on similar timescales was assigned to intraprotein EET. Although we have no direct evidence to rule out the possibility that this timescale corresponds to EET from CP43 to RC, it is more likely that it reflects the EET within CP43. The 1,691-cm^−1^ peak was assigned to a blue Chl in the study of isolated CP43 by Di Donato et al. (52). This explains why the spectral feature was not observed in the first group, where the ω_exc_ range is lower. In addition to the 1,691 cm^−1^ peak and the PIA spanning 1,710 to 1,760 cm^−1^, evolution around the PIA peak at 1,654 cm^−1^ is also found in this step (Fig. 3*C*, evolution from red to yellow EADS). This peak undergoes a clear decay, whereas the intensity and the structure of the remainder of the PIA band (spanning 1,620 to 1,660 cm^−1^) remains relatively constant. The decay of this 1,654 cm^−1^ peak was observed in both isolated CP43 and CP47, further indicating that the first step is dominated by EET within in the antenna complexes

The second step (Fig. 3*C*, evolution from yellow to green EADS) exhibits a further decay of the 1,691 cm^−1^ peak on a timescale of ∼1.8 ps. The continued decay of the 1,691-cm^−1^ peak suggests that an additional EET pathway is captured in this component. Interestingly, the evolution of the 1,691-cm^−1^ peak on this timescale was not observed in previous work by Di Donato et al. (52) on isolated CP43 . In contrast to the first timescale (∼180 fs), which may not have been resolved due to time resolution and/or exciton–exciton annihilation, the lack of this timescale in isolated CP43 strongly suggests that this step involves EET from CP43 to the RC, most likely involving the peripheral pigment Chlz_D1_, which will be discussed in further detail later. The final step (Fig. 3*C*, green to blue EADS) resembles that of the first group (Fig. 3*B*); however, the timescale is noticeably longer. This is actually consistent with what was observed in the isolated RC (45). At higher ω_exc_, the CS dynamics are limited by EET from the peripheral Chlz_D1_ pigment of the RC into the electron transfer-active D1 branch pigments. We attribute the discrepancies in the observed timescale for complete CS in the PSII-CC (∼9.76 ps) versus previous studies for the isolated PSII-RC (14-37 ps) (16, 59) to contributions from concurrent EET pathways within the PSII-CC that can influence the obtained time constants.

Finally, for the third group (Fig. 3*D*, representative ω_exc_ = 15,156 cm^−1^), three components were needed to obtain reasonable fits. Despite the fact that the number of components needed is the same as the first group, we choose to distinguish between these two groups because of their clear energetic separation, which we expect to give rise to distinct dynamics because excitonic states of very different character are populated in these two regions. Similar to the second group, the first step here (Fig. 3*D*, evolution from red to green EADS) shows a clear decay of the 1,691-cm^−1^ peak and an accompanying growth of the PIA band spanning 1,710 to 1,760 cm^−1^. The distinction here, though, is that the 1,691-cm^−1^ peak has almost completely decayed on this timescale (∼480 fs). This suggests that the two EET pathways discussed above (intraprotein EET and EET from CP43 to RC) are convoluted together in this region and could not be distinguished. The final step in this group (Fig. 3*D*, green to blue EADS) closely resembles the final step from the second group and is likewise assigned to complete CS limited by EET within the RC.

It is worth noting that we did not observe a net decay spanning throughout the entire ω_det_ axis in any of the EADS, which was present in the previous transient IR experiments for CP43, CP47, and PSII-CC (19, 50, 52) and assigned to exciton–exciton annihilation. This is a clear indication that exciton–exciton annihilation is not significant under our excitation condition as intended. Additionally, we did not observe the longer timescales (30∼50 ps) that were present in other experiments (15, 16, 19–21). This is possibly due to weaker signals at later waiting times in our experiment, which causes these timescales to be convoluted with other faster processes. Moreover, the spectral markers for CS are particularly strong and could obscure other features in the same spectral range. Finally, due to the accumulation of charges, the RC in our experiment stayed closed, i.e., the secondary electron acceptor, quinone, remained reduced (Q_A_^−^). Under this condition, the primary CS still occurs (21, 60), and the CS species will decay through charge recombination, resulting in triplet states that decay in microsecond timescales (61, 62), shorter than the repetition rate in our experiment (1 kHz). Therefore, as we focus on the sub-100-ps dynamics, we do not expect the accumulation of charges in the RC pigments other than Q_A_. It was previously suggested that CS in the closed RC is slowed down compared to the open RC (21, 22). Interestingly, we did not observe such a phenomenon when comparing the current results with the work on isolated RC (in which no Q_A_ was present) (45). This is consistent with the recent 2DES experiments on PSII-CC with open and closed RC, in which Akhtar et al. (60) observe excitation decay in 3∼5 ps under both conditions. However, a comparison between the 2DEV experiments on the isolated RC and the PSII-CC remains difficult as the difference could also be due to structural variation of the RC when isolated versus attached to the core antennas.

## Discussion

### A Detailed Scheme for the Dynamics of the PSII-CC.

The representative global analyses from each group have already provided a basic scheme to describe the dynamics. Here, we will develop a more explicit understanding of the EET and CS processes within each group based on the differences between the timescales and the structure of the EADS (whereas the criteria for the separation into groups are based on the number of components required for fitting).

**Fig. 4.**
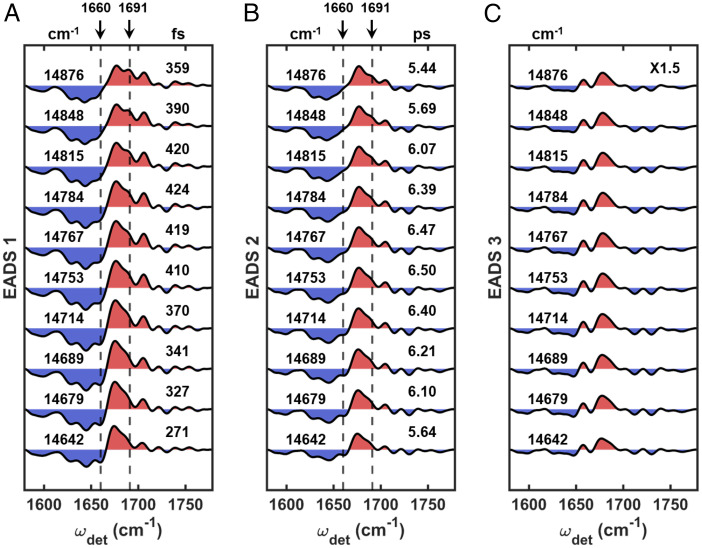
Comparison of the (*A*) first, (*B*) second, and (*C*) third (scaled by a factor of 1.5) EADS in the first group. In each panel, ω_exc_s are shown on the *Left* of the plots and the corresponding time constants are shown on the *Right* (with units above). The last component in the fit is a nondecaying component and therefore has no corresponding time constants. The SEs of the time constants are all within 1% to 5%. The specific values are listed in *SI Appendix*, Tables S1–S3. Throughout, features shaded red and blue indicate GSBs and PIAs, respectively.

Fig. 4 shows a direct comparison for the global analyses spanning the entire ω_exc_ range for the first group. Although the dominant contribution to the dynamics in this group arises from CS, we can see some differences in both the structure of the EADS and the timescales throughout the group. For example, in the first EADS (Fig. 4*A*), the marker for CS around 1,660 cm^−1^ becomes less prominent as ω_exc_ increases, while the characteristic peak for CP43 at 1,691 cm^−1^ emerges more clearly at higher ω_exc_. These trends as a function of increasing ω_exc_ result from the following: 1) a decreasing degree of exciton–CT mixing throughout the RC excitonic manifold (45, 55) and 2) increased excitation of the states in the excitonic manifold of CP43. The time constants for the first step, shown in Fig. 4*A*, are also consistent with there being varying degrees of contribution from CS and EET dynamics throughout this ω_exc_ range. For a ω_exc_ lower than 14,815 cm^−1^, the time constants become longer as ω_exc_ increases. This is as expected because less exciton–CT mixing should result in a longer CS time (45). Above 14,815 cm^−1^, EET within CP43 (reflected by the 1,691-cm^−1^ peak) should begin to influence the time constants such that they become shorter again because EET is a faster process (∼180 fs estimated from the dynamics of the second group in the previous section) than CS (300∼400 fs). Therefore, the trend of the time constants further consolidates that, at higher ω_exc_ in this group, the contribution of EET to the observed dynamics becomes more prominent (in addition to the dominant pathway of CS within the RC). The ω_exc_ at which the 1,691-cm^−1^ peak starts to appear (∼14,848 cm^−1^) is very similar in energy to the excitonic states of CP43 that are mostly localized on pigment C512 or C508 (14,852 cm^−1^ and 14,907 cm^−1^, respectively, based on the empirical Hamiltonian introduced by Müh et al. (56)), suggesting that the 1,691-cm^−1^ peak could arise from either or both of these Chls. From the comparison shown in Fig. 4*A*, it is also clear that the emergence of the peak at 1,691 cm^−1^ with increasing ω_exc_ begins to overshadow the shoulder at 1,686 cm^−1^, which again, limits the understanding of CP47 dynamics.

The second EADS in the first group (Fig. 4*B*) generally have the same structure as a function of ω_exc_ that makes it difficult to definitively understand the origin of the trend in the time constants. Minor difference can be found, however, around 1,660 cm^−1^ and 1,691 cm^−1^. As the trends in this second step generally follow those of the first step, varying contributions from parallel CS and EET pathways likely cause the observed ω_exc_ dependence. For example, concurrent contributions from the slower pathway of EET from CP43 to the RC (∼1.8 ps estimated from the dynamics of the second group) could play a role in the observed trend at higher ω_exc_ due to the simultaneous excitation of CP43 and the RC. This is likely especially as the excitonic manifold becomes highly congested in this ω_exc_ range.

**Fig. 5.**
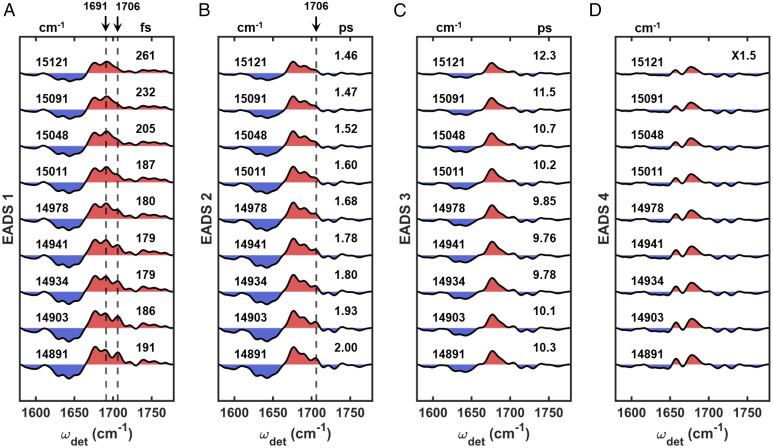
Comparison of the (*A*) first, (*B*) second, (*C*) third, and (*D*) fourth (scaled by a factor of 1.5) EADS in the second group. In each panel, ω_exc_s are shown on the *Left* of the plots and the corresponding time constants are shown on the *Right* (with units above). The last component in the fit is a nondecaying component and therefore has no corresponding time constants. The SEs of the time constants are all within 1% to 5%. The specific values are listed in *SI Appendix*, Tables S1–S3. Throughout, features shaded red and blue indicate GSBs and PIAs, respectively.

The final EADS (Fig. 4*C*, scaled by a factor of 1.5) appear to be identical to each other within this group. In fact, they remain the same along the entirety of the ω_exc_ axis (Fig. 5*D* and *SI Appendix*, Fig. S2*C*). Since the final component in all of the fits is nondecaying for the duration of the experiment, the similarity indicates that the same final state, Pheo_D1_^−^(P_D1_P_D2_)^+^, is reached regardless of the initial ω_exc_.

The comparison of the second group is shown in Fig. 5. In general, the dynamics for this group is characterized by two EET pathways followed by CS. The dynamics in this region is more complex than for the first group, which is reflected in the multiple trends for the time constants. As a result, we mainly focus on the trends in the spectral structure of the EADS that are more straightforward to interpret than the trends for the time constants. In the first EADS (Fig. 5*A*), the intensities for both the characteristic peak of CP43 at 1,691 cm^−1^ and the characteristic marker for the RC at 1,706 cm^−1^ are noticeably ω_exc_ dependent. This indicates that there are varying degrees of contribution from excitonic states of the RC versus CP43. For example, the peak at 1,706 cm^−1^ loses intensity as ω_exc_ approaches the top of the RC manifold around 15,034 cm^−1^. On the other hand, the feature at 1,691 cm^−1^ reaches a maximum intensity around 14,978 cm^−1^. This ω_exc_ is also approximately where the absorption maximum of CP43 occurs (26, 63, 64). The CP43 character clearly dominates in the ω_exc_ region above 14,978 cm^−1^ (whereas there are more substantial contributions from the RC below this ω_exc_ threshold).

The ω_exc_ trend for the second EADS is shown in Fig. 5*B*. Although the dominant pathway for this step is assigned to EET from CP43 to the RC, when analyzing the intragroup trends, we observe differences in the EADS around 1,706 cm^−1^, the marker for the RC. This could indicate the presence of a parallel EET pathway within the RC manifold. In this case, the intensity of this mode seems to be correlated to the trend in the time constants that become shorter as the intensity decreases. This could indicate that EET from CP43 to the RC is faster than dynamical processes within the RC because CP43 character dominates the higher ω_exc_ region. Regarding the origin of the pathway of EET from CP43 to the RC, both the spatial arrangement of the pigments in the PSII-CC and the corresponding energetic landscape point to the likely involvement of excitonic states of mainly C505 character. C505 in CP43 is spatially the closest pigment to the peripheral Chlz_D1_ pigment on the D1 side of the RC (center-to-center distance, ∼21 Å), and the energies of these excitonic states, which fall within the range of this group, are close to each other (15,011 cm^−1^ for C505, 14,941 cm^−1^ for Chlz_D1_). From the comparison within the first group, we established that the 1,691-cm^−1^ peak likely originates from C512 and/or C508, of which both are on the stromal side. Because C505 is also on the stromal side, fast equilibration could occur, especially with C508 where the center-to-center distance is ∼15 Å. This suggests that the further decay of the 1,691-cm^−1^ peak can also arise from the population decay of C505. In this case, EET from C505 to Chlz_D1_ (∼1.8 ps), reflected by the decay of the 1,691-cm^−1^ peak, is faster than EET from Chlz_D1_ to other pigments in the D1 branch (∼10 ps). This is consistent with previous suggestions that Chlz_D1_, although bound to the D1 protein, is functionally closer to the antennas (22).

The third EADS (Fig. 5*C*) and fourth EADS (Fig. 5*D*) do not show any apparent ω_exc_ dependence. There is some ω_exc_ dependence in the time constants, but this is likely due to a varying degree of convolution between the processes following the two major pathways of EET from the antenna, which cannot be completely untangled with the current model.

The comparison for the third and final group is shown in the *SI Appendix*, Fig. S3 as the fewer number of states in this range does not support an in-depth analysis of the intragroup trends. In addition, it is difficult to assign the origin of this pathway because the observed timescales do not clearly follow the two EET pathways that involve the evolution of the 1,691-cm^−1^ peak described above for the second group. However, the final step in this group, which has nearly identical timescales and EADS structure with the final step in the second group, hints that similar EET from CP43 to RC also occurs at this ω_exc_ range so that the subsequent dynamics in the RC are similar.

**Fig. 6.**
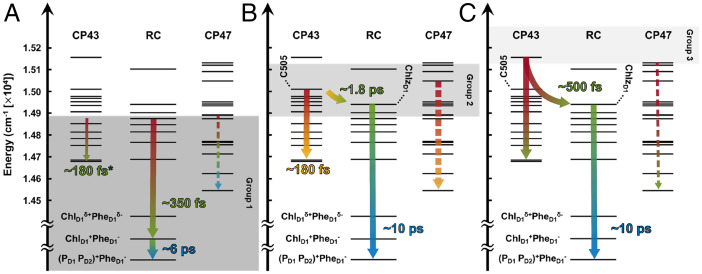
Schematic diagram summarizing the characteristic dynamics of the PSII-CC displayed by the (*A*) first, (*B*) second, and (*C*) third group. The arrows are colored according to Fig. 3. Throughout, dominant pathways for each group are shown with thicker arrows and contributing pathways discerned through an analysis of intragroup trends are represented by thinner arrows with the corresponding timescales marked by an asterisk (as they can only be inferred from a different group). The CP47 dynamics are represented with dotted arrows, and the timescales are not shown due to the lack of clear evidence. Excitonic levels arising from particularly important pigments are labeled. The different directions for the arrows with the same origin indicate the existence of uncertainty for the observed dynamical processes.

We summarize the observed sub-100-ps dynamics of the PSII-CC at 77 K resulting from our analysis in Fig. 6. Overall, the dynamics largely reflect either 1) intraprotein dynamics or 2) EET from CP43 to the pigments on the D1 branch of the RC. We stress that the CP47 dynamics shown in Fig. 6 (represented by dotted arrows) are only tentative assignments that arise from the limited evidence associated with CP47 in the spectra. Within each group, dominant pathways (thicker arrows) were assigned through the observed vibrational structure of the EADS and corresponding timescales, whereas the presence of additional pathways (thinner arrows) was untangled through the analysis of intragroup trends. For the first group (Fig. 6*A*), rapid CS ((Chl_D1_^δ+^Pheo_D1_^δ−^)* → Chl_D1_^+^Pheo_D1_^−^) from the energetically lower-lying excitonic levels of the RC is followed by hole transfer from Chl_D1_^+^ to (P_D1_P_D2_)^+^ (45). For the first step in this group, participation of EET within CP43 also begins to contribute to the observed dynamics (indicated by the thinner arrows in Fig. 6*A*)—particularly at higher excitation frequencies. EET within CP47 can also occur on similar timescales in this group. In the second group (Fig. 6*B*), the participation of the EET within CP43 is much more apparent, and an additional EET channel from CP43 to the RC is observed, which likely involves the C505 state of CP43 and the peripheral Chlz_D1_ pigment of the RC. Following this interprotein EET step, the dominant dynamics observed is CS in the RC limited by EET. For the third and final group (Fig. 6*C*), specific assignments remain difficult; however, the results of the global analyses suggest EET from CP43 to the RC is similar to the second group and is followed by EET-limited CS within the RC as well. Finally, it is worth mentioning again that the lack of an observation of CP47 dynamics is because of the low temperature at which the experiment was conducted, as suggested by the prediction of structure-based calculations (22) and temperature-dependent fluorescence studies (26).

### Concluding Comments.

Here, we have demonstrated the ability of 2DEV spectroscopy to provide detailed information about the dynamics in complex photosynthetic systems. Specifically, we have shown that the initial, ultrafast dynamics in the PSII-CC at 77 K are highly dependent on the ω_exc_, i.e., the interplay between intraprotein dynamics and interprotein EET varies drastically in different energetic regions. In the lower energetic region, the dominant dynamics observed are intraprotein dynamics. The spectral evolution in this range shows clear signatures for CS in the RC, allowing the direct extraction of CS information from the 2DEV spectra without the issue of being overshadowed by the complex EET scheme that would be triggered at higher ω_exc_. In the higher energetic region, EET from CP43 to RC is observed. The overall dynamics in this ω_exc_ range resembles the TTTL model, where CS is limited by EET (*k*_CS_ > *k*_EET_). The 2DEV spectral evolution provides EET information in two regards, as follows: 1) C505 and Chlz_D1_ are within the ω_exc_ range in which EET is observed and 2) the vibrational structure indicates EET out of CP43. The combination of both suggests that EET from CP43 to Chlz_D1_ is faster than EET from Chlz_D1_ to other D1 pigments, and the latter is the actual kinetic bottleneck. Interestingly, this does not contradict the TTTL model, in which the rate-determining step is the EET from the antenna systems to the RC. In fact, this provides evidence that supports the previous assumptions that functionally Chlz_D1_ belongs to the antenna system. The ability to experimentally obtain such detailed information shows that the dual resolution afforded by 2DEV spectroscopy is critical in the studies of complex systems.

In addition, we noted earlier that global analyses of the evolution of time-resolved spectra often require a reduced kinetic scheme, which does not necessarily describe the dynamics in complex systems. For example, for the PSII-CC, where entropic and energetic factors likely determine the overall ET and CS timescales, the application of a sequential model that contains only unidirectional steps may limit the insight available into, for example, the roles of the various protein subunits or where bottlenecks in the energy flow lie. Previously, to overcome such limitations, a formalism was developed by Yang and Fleming (65) and later applied by Bennett et al. (25) Starting with a full rate matrix for the system under study, the method allows the construction of physically and kinetically distinct domains. Then, the energy flow is described as a sequence of steps from higher order domains to the lowest order domain. This process is rigorous and unique for a given rate matrix. With the improved ability of 2DEV spectroscopy to separate parallel pathways, connecting global analysis results and the description of such formalism could provide substantially improved insight into the pathways of energy flow in PSII-CC and perhaps in larger subunits of the PSII-RC/antenna system. This allows further extension of the current kinetic models. For example, one could construct a model that includes a coarse-grained energy landscape that reflects the characteristic dynamics in different energetic regions as observed here. Such improvement will allow the model to contain not only structural but also energetic information, setting a step forward in the path of understanding the design principal of photosynthetic systems. We believe this work provides a solid foundation for the future studies of larger systems, which have more complicated networks and more robust functions.

## Materials and Methods

### Isolation of the PSII-CC.

All procedures for sample preparation were performed in the dark to minimize exposure to light as much as possible. We prepared PSII-enriched membranes according to the previous literature (66, 67) with some modifications as described previously (45). We isolated PSII-CC according to the previous literature (68) with some modifications as follows. The PSII-enriched membranes (0.5 mg Chl/mL) were solubilized with 0.5% (wt/vol) n-octyl-β-D-thioglucoside (Anatrace) in a buffer containing 50 mM MES-NaOH (pH 6.0), 10 mM NaCl, and 400 mM sucrose for 10 min on ice. The solution was centrifuged at 40,000 × g for 30 min at 4 °C. The supernatant was collected and diluted with the same buffer at a 1:1.2 ratio. Then, the final concentration of 10 mM MgCl_2_ was added and mixed on ice for 5 min. The mixture was centrifuged at 40,000 × g for 10 min at 4 °C. The supernatant was collected and mixed with a buffer containing 50 mM MES-NaOH (pH 6.0) and 20% (wt/vol) polyethylene glycol 6000 at a 1:1 ratio. The mixture was centrifuged at 40,000 × g for 30 min at 4 °C. The pelleted PSII-CC was washed with a buffer containing 50 mM MES-NaOH (pH 6.0), 10 mM NaCl, 3 mM CaCl_2_, and 400 mM sucrose and centrifuged at 40,000 × g for 10 min at 4 °C. The PSII-CC was resuspended with the same buffer except that H_2_O was replaced with D_2_O and centrifuged at 15,000 × g for 15 min at 4 °C. The pelleted PSII-CC was resuspended with the same buffer prepared with D_2_O and solubilized with the final concentration of 0.8% (wt/vol) n-dodecyl-β-D-maltoside (Anatrace). The PSII-CC was flash frozen and stored at −80 °C until 2DEV measurements were performed.

### 2DEV Spectroscopy.

The output of a Ti:Sapphire oscillator (Vitara-S, Coherent) and regenerative amplifier (Legend, Coherent) was used to generate the visible and mid-IR pulses with a home-built noncollinear optical parametric amplifier (NOPA) and optical parametric amplifier–difference frequency generation instrument, respectively. The output of the NOPA (centered at 680 nm with full-width half maximum 65 nm) was compressed to ∼22 fs with a prism pair and a pulse shaper (Dazzler, Fastlite). The visible pulses had a combined energy of ∼80 nJ. The pulses were focused on the sample to a spot size of ∼250 μm. To compensate the temporal dispersion of the mid-IR pulse, 9 mm of Ge plates were used. Cross-correlation between the visible and mid-IR pulses recovered a ∼90-fs instrument response function with a 50-μm Ge plate. The mid-IR pulse was split by a 50:50 beam splitter to produce a probe and a reference beam. Both beams were focused on the sample to a spot size of ∼200 μm. After passing through the sample, the mid-IR beams were dispersed with a spectrometer (Triax 180, Horiba) onto a dual-array 64-pixel HgCdTe detector (Infrared Systems Development). The delay between the two visible pulses *t*_1_ was scanned (using the pulse shaper) from 0 to 100 fs in ∼2.4-fs time steps. For each *t*_1_, the desired signal was isolated with a 3 × 1 phase cycling scheme (69, 70). The excitation axis was obtained by performing Fourier transform along the *t*_1_ axis. The waiting time, *T*, controlled by a motorized translation stage, was scanned from −100 to 1,050 fs in 25-fs time steps, 1,150 to 5,150 fs in 100-fs steps, 5,650 to 20,150 fs in 500-fs steps, and 30,000 to 100,000 fs in 10,000-fs time steps, for a total of 125 steps. For the measurement, the PSII-CC sample was mixed with glycerol-d_8_ (8:2 [vol/vol], glycerol-d_8_:PSII-CC) and kept at 77 K in an optical cryostat (OptistatDN2, Oxford Instruments). The maximum optical density in the visible excitation range was ∼1.0 at 670 nm with an optical path length of ∼200 μm.

## Supplementary Material

Supplementary File

## Data Availability

All study data are included in the article and/or *SI Appendix*. The data presented in this study are available from the corresponding author upon request.
