# Supplementary material for: From antenna to reaction center: Pathways of ultrafast energy and charge transfer in photosystem II
Source: Proc Natl Acad Sci U S A. 2022 Oct 10;119(42):e2208033119. doi: 10.1073/pnas.2208033119 (PMC9586314; doi:10.1073/pnas.2208033119)
Supplement: Supplementary File [file pnas.2208033119.sapp.pdf]

Supplementary Information for

From antenna to reaction center: Pathways of ultrafast energy and charge transfer in photosystem II

Shiun-Jr Yang[1,2], Eric A. Arsenault[1,2,3], Kaydren Orcutt[1,2], Masakazu Iwai[2,4], Yusuke Yoneda[5], and Graham R. Fleming[1,2,3*]

[1]*Department of Chemistry, University of California, Berkeley, CA 94720, USA*

[2]*Molecular Biophysics and Integrated Bioimaging Division, Lawrence Berkeley National Laboratory, Berkeley, CA 94720, USA*

[3]*Kavli Energy Nanoscience Institute at Berkeley, Berkeley, CA 94720, USA*

[4]*Department of Plant and Microbial Biology, University of California, Berkeley, CA 94720, USA*

[5]*Research Center of Integrative Molecular Systems, Institute for Molecular Science, National Institute of Natural Sciences, Okazaki, Aichi, 444-8585, Japan*

*grfleming@lbl.gov

This PDF file includes:

Supplementary text
Figures S1 to S2
Tables S1 to S3
SI References

Supplementary Information Text

Global Analysis. Global analyses were performed using Glotaran (1) on individual 2DEV slices at fixed ω_{exc}. The 2DEV slices were fitted with sequential models that contain three and four components, where the last component in both models is set as non-decaying. The complete set of the results can be found in Tables S1 to S3 which includes the fitted rates and time constants as well as associated errors. The time constant errors were calculated from the propagation of the rate errors. As shown in the tables, the trends in the time constants discussed in the main manuscript are not within error, allowing for a comparison.

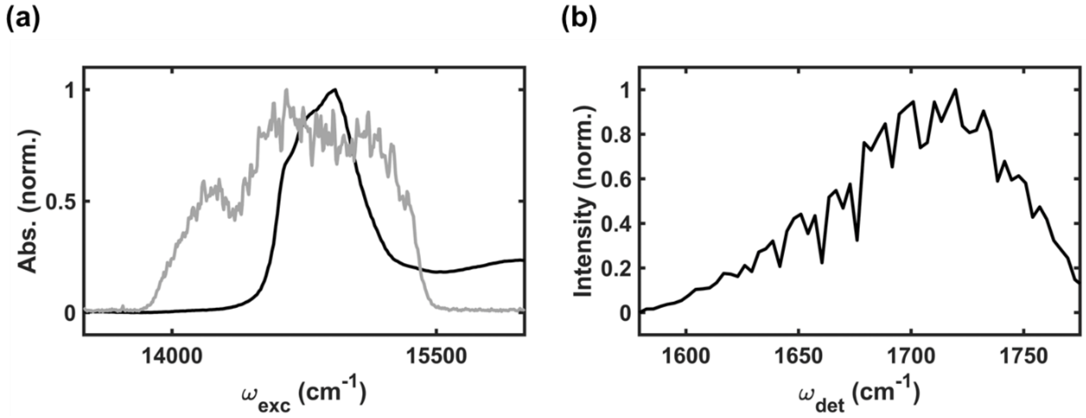

Fig. S1. (a) Normalized linear absorption spectrum of the PSIICC at 77K (black) and the spectrum of the visible excitation pulses (grey). (b) Normalized spectrum of the IR detection pulse.

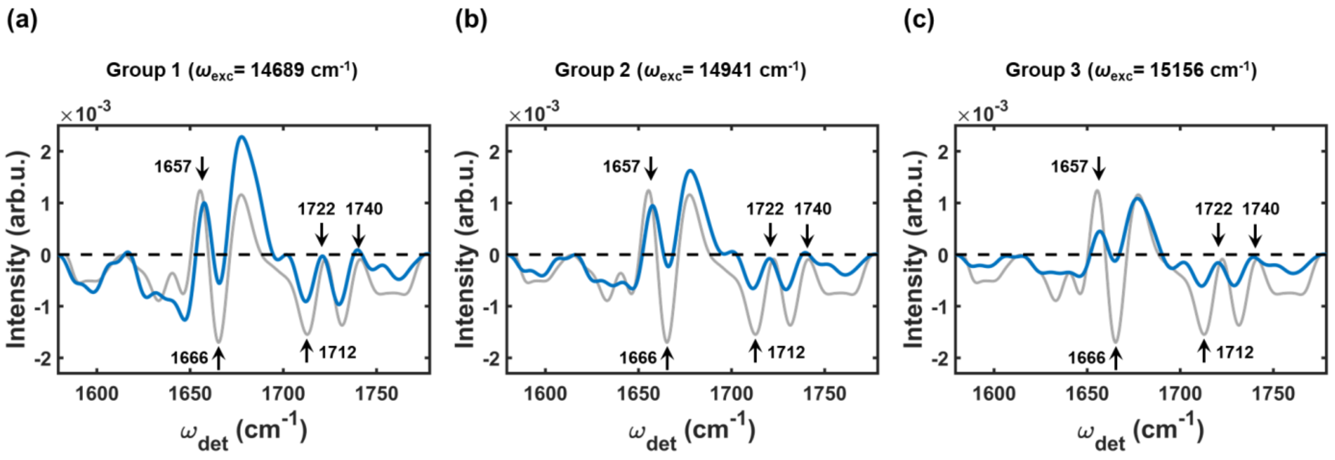

Fig. S2. Comparison of the final EADS (blue) with the 2DEV slice (ω_{exc}=14689 cm^{-1} (exciton 2); 89 ps) of isolated RC (grey) for the representative ω_{exc} of each group (2). The peaks with labels are the key identifier for the charge separated state $(P_{D1}P_{D2})^{+}Pheo_{D1}^{-}$. The isolated RC 2DEV slice was reproduced with permission from Yoneda et al. Nat Comm 13, 2275 (2022). Copyright 2022 Author(s) licensed under a Creative Commons Attribution 4.0 Licence.

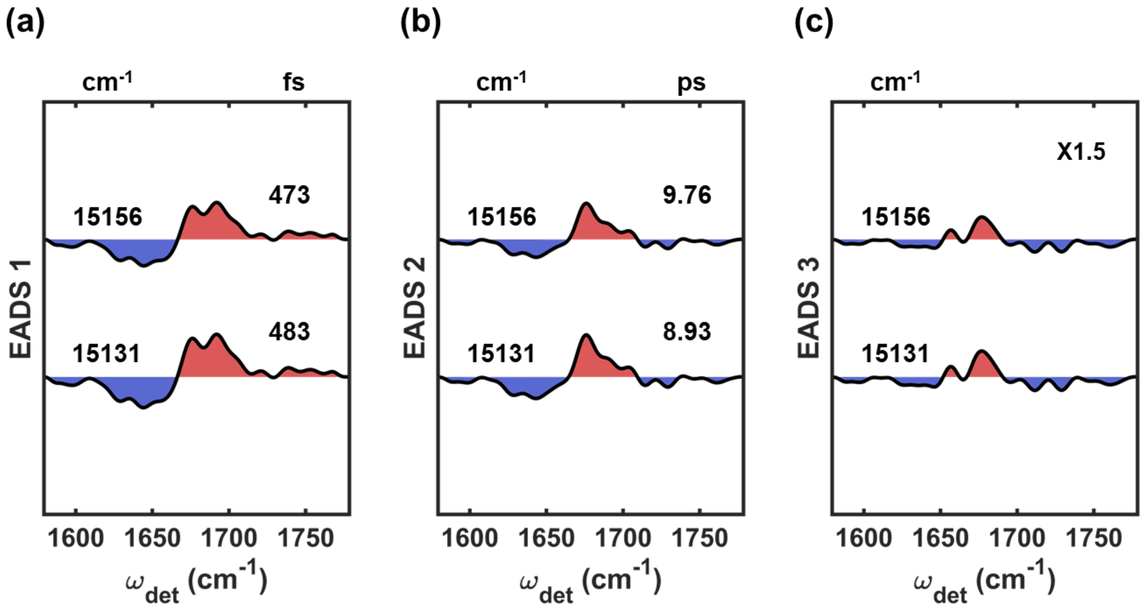

Fig. S3. Comparison of the (a) first, (b) second, and (c) third (scaled by a factor of 1.5) EADS in the third group. In each panel, ω_{exc} are shown on the left of the plots and the corresponding time constants are shown on the right (with units above). The last component in the fit is a non-decaying component and therefore has no corresponding time constants. The standard errors of the time constants are all within 1-5%. The specific values are listed in table S1-S3. Throughout, features shaded red (blue) indicate GSBs (PIAs).

Table S1. First components in the global analyses at all energetic levels.

	Energy Level (cm^{-1})	Rate (fs^{-1})	Rate Error (fs^{-1})	Time Constant (fs)	Time Constant Error (fs)
Group 1	14428	0.00472	0.00013	212	6
	14546	0.00488	0.00008	205	3
	14624	0.00369	0.00006	271	5
	14679	0.00306	0.00005	327	6
	14686	0.00293	0.00005	341	6
	14689	0.00293	0.00005	341	6
	14714	0.00270	0.00005	370	6
	14753	0.00244	0.00004	410	7
	14756	0.00244	0.00004	410	7
	14767	0.00239	0.00004	419	8
	14772	0.00239	0.00004	419	8
	14784	0.00236	0.00004	424	8
	14814	0.00238	0.00005	420	8
	14815	0.00238	0.00005	420	8
	14848	0.00256	0.00005	390	8
	14853	0.00261	0.00005	383	7
	14875	0.00279	0.00005	359	7
	14876	0.00279	0.00005	359	7
Group 2	14891	0.00523	0.00010	191	4
	14893	0.00530	0.00011	189	4
	14903	0.00537	0.00011	186	4
	14907	0.00543	0.00011	184	4
	14934	0.00558	0.00011	179	4
	14936	0.00558	0.00011	179	4
	14941	0.00559	0.00011	179	4
	14943	0.00559	0.00011	179	4
	14953	0.00560	0.00011	179	4
	14969	0.00558	0.00011	179	4
	14978	0.00556	0.00011	180	4
	15011	0.00534	0.00011	187	4
	15048	0.00488	0.00010	205	4
	15091	0.00430	0.00008	232	4
	15103	0.00411	0.00008	243	5
	15121	0.00383	0.00007	261	5
3	15131	0.00211	0.00003	473	7
	15156	0.00207	0.00003	483	8

Table S2. Second components in the global analyses at all energetic levels.

	Energy Level (cm^{-1})	Rate (fs^{-1})	Rate Error (fs^{-1})	Time Constant (fs)	Time Constant Error (fs)
Group 1	14428	0.000390	0.000011	2570	70
	14546	0.000186	0.000004	5360	120
	14624	0.000177	0.000004	5640	120
	14679	0.000164	0.000003	6100	130
	14686	0.000161	0.000003	6210	130
	14689	0.000161	0.000003	6210	130
	14714	0.000156	0.000003	6400	130
	14753	0.000154	0.000003	6500	130
	14756	0.000154	0.000003	6500	130
	14767	0.000155	0.000003	6470	130
	14772	0.000155	0.000003	6470	130
	14784	0.000156	0.000003	6390	120
	14814	0.000165	0.000003	6070	110
	14815	0.000165	0.000003	6070	110
	14848	0.000176	0.000003	5690	90
	14853	0.000178	0.000003	5620	90
	14875	0.000184	0.000003	5440	80
	14876	0.000184	0.000003	5440	80
Group 2	14891	0.000501	0.000014	2000	50
	14893	0.000509	0.000014	1960	50
	14903	0.000517	0.000014	1930	50
	14907	0.000525	0.000015	1900	50
	14934	0.000555	0.000016	1800	50
	14936	0.000555	0.000016	1800	50
	14941	0.000562	0.000016	1780	50
	14943	0.000562	0.000016	1780	50
	14953	0.000575	0.000016	1740	50
	14969	0.000588	0.000017	1700	50
	14978	0.000595	0.000017	1680	50
	15011	0.000626	0.000018	1600	50
	15048	0.000658	0.000019	1520	40
	15091	0.000679	0.000021	1470	40
	15103	0.000683	0.000021	1460	50
	15121	0.000685	0.000022	1460	50
3	15131	0.000112	0.000002	8940	170
	15156	0.000103	0.000002	9760	210

Table S3. Third components in the global analyses at group 2 energetic levels.

	Energy Level (cm^{-1})	Rate (fs^{-1})	Rate Error (fs^{-1})	Time Constant (fs)	Time Constant Error (fs)
Group 2	14891	0.0000972	0.00000238	10300	300
	14893	0.0000983	0.00000239	10200	200
	14903	0.0000993	0.00000239	10100	200
	14907	0.0001002	0.00000239	10000	200
	14934	0.0001022	0.00000239	9780	230
	14936	0.0001022	0.00000239	9780	230
	14941	0.0001024	0.00000238	9760	230
	14943	0.0001024	0.00000238	9760	230
	14953	0.0001024	0.00000237	9770	230
	14969	0.0001019	0.00000236	9810	230
	14978	0.0001015	0.00000235	9850	230
	15011	0.0000985	0.00000231	10200	200
	15048	0.0000933	0.00000228	10700	300
	15091	0.0000872	0.00000226	11500	300
	15103	0.0000851	0.00000226	11800	300
	15121	0.0000816	0.00000226	12300	300

SI References

1. J. J. Snellenburg, S. Laptenok, R. Seger, K. M. Mullen, I. H. M. van Stokkum, Glotaran: A Java-Based Graphical User Interface for the R Package TIMP. *Journal of Statistical Software* **49**, 1 - 22 (2012).
2. Y. Yoneda *et al.*, The initial charge separation step in oxygenic photosynthesis. *Nature Communications* **13**, 2275 (2022).
